# Processing and Properties of Polyhydroxyalkanoate/ZnO Nanocomposites: A Review of Their Potential as Sustainable Packaging Materials

**DOI:** 10.3390/polym16213061

**Published:** 2024-10-30

**Authors:** Mieke Buntinx, Chris Vanheusden, Dries Hermans

**Affiliations:** 1 Materials and Packaging Research & Services (MPPR&S), Institute for Materials Research (Imo-Imomec), Hasselt University, Martelarenlaan 42, B-3500 Hasselt, Belgium; chris.vanheusden@uhasselt.be (C.V.); dries.hermans@uhasselt.be (D.H.); 2 Imec, Imo-Imomec, Wetenschapspark 1, B-3590 Diepenbeek, Belgium

**Keywords:** polyhydroxyalkanoates, PHA, ZnO nanoparticles, solvent casting, melt processing, centrifugal fiber spinning, nanoparticle encapsulation, coating, active packaging

## Abstract

The escalating environmental concerns associated with conventional plastic packaging have accelerated the development of sustainable alternatives, making food packaging a focus area for innovation. Bioplastics, particularly polyhydroxyalkanoates (PHAs), have emerged as potential candidates due to their biobased origin, biodegradability, and biocompatibility. PHAs stand out for their good mechanical and medium gas permeability properties, making them promising materials for food packaging applications. In parallel, zinc oxide (ZnO) nanoparticles (NPs) have gained attention for their antimicrobial properties and ability to enhance the mechanical and barrier properties of (bio)polymers. This review aims to provide a comprehensive introduction to the research on PHA/ZnO nanocomposites. It starts with the importance and current challenges of food packaging, followed by a discussion on the opportunities of bioplastics and PHAs. Next, the synthesis, properties, and application areas of ZnO NPs are discussed to introduce their potential use in (bio)plastic food packaging. Early research on PHA/ZnO nanocomposites has focused on solvent-assisted production methods, whereas novel technologies can offer additional possibilities with regard to industrial upscaling, safer or cheaper processing, or more specific incorporation of ZnO NPs in the matrix or on the surface of PHA films or fibers. Here, the use of solvent casting, melt processing, electrospinning, centrifugal fiber spinning, miniemulsion encapsulation, and ultrasonic spray coating to produce PHA/ZnO nanocomposites is explained. Finally, an overview is given of the reported effects of ZnO NP incorporation on thermal, mechanical, gas barrier, UV barrier, and antimicrobial properties in ZnO nanocomposites based on poly(3-hydroxybutyrate), poly(3-hydroxybutyrate-co-3-hydroxyvalerate), and poly(3-hydroxybutyrate-co-3-hydroxyhexanoate). We conclude that the functionality of PHA materials can be improved by optimizing the ZnO incorporation process and the complex interplay between intrinsic ZnO NP properties, dispersion quality, matrix–filler interactions, and crystallinity. Further research regarding the antimicrobial efficiency and potential migration of ZnO NPs in food (simulants) and the End-of-Life will determine the market potential of PHA/ZnO nanocomposites as active packaging material.

## 1. Challenges of Plastic (Food) Packaging Technology

The primary role of food packaging is to preserve food, to ensure food quality and safety, and to avoid food waste. Historically, extending the shelf life of food products has relied on controlling the transfer of gasses and contaminants using packaging materials, in ancient times through natural materials such as clay in pottery, and later, in the industrial era, using glass and metal [1,2]. However, the increase in the diversity of food products and their consumption led to the development of modern packaging technologies, in which plastics play an essential role [3]. Plastics are generally characterized by high strength-to-weight ratios, low costs, and easy processing while having the possibility of being transparent or opaque, and rigid or flexible. Plastics are significantly lighter and cheaper for mass production than glass or metal, reducing transportation and production costs [4]. Due to this versatility, worldwide plastic production keeps increasing, especially in the packaging industry, which has the highest demand for plastics on a global scale. The most commonly used packaging materials are low- and high-density polyethylene (LDPE and HDPE), polypropylene (PP), polyethylene terephthalate (PET), polystyrene (PS), and polyvinylchloride (PVC) [5].

An example of a widely used packaging technology is modified atmosphere packaging (MAP). Here, the atmosphere (e.g., O_2_, N_2_, and CO_2_) inside the packaging is adapted to reduce undesirable physicochemical changes in food, to control microbial growth, and to increase shelf life. However, to maintain a certain atmosphere, the packaging materials used require specific gas permeability properties in combination with sufficient strength, flexibility, and seal quality [6]. In most cases, it is impossible to obtain the required properties by using a single monolayer polymer, but this is solved by multilayer packaging, which combines various plastic layers with different unique properties. This is ideal for material reduction, although they are currently difficult to recycle [7].

Unfortunately, food waste is still a huge challenge in the food industry. In 2020, food waste in the EU was estimated at ~59 million tons of fresh mass, with a 53% share of household food waste [8]. This and present-day drivers such as the consumer’s demand for healthy, fresh, minimally processed, safe, and high-quality foods with a long shelf life [9], have led to the development of novel packaging technologies such as smart packaging. Smart packaging technologies include both active and intelligent concepts. Active packaging (AP) aims to maintain the product quality and extend the shelf life by interacting with the product in a positive way, while intelligent packaging (IP) aims at communicating specific information or the quality of the packaged product by monitoring the condition of the product [10]. AP technologies include oxygen and ethylene scavengers, antibacterial agents, and moisture absorbers [11]. IP technologies include indicators and sensors to detect or monitor humidity, pH, temperature, chemicals, or bacteria [12]. Current research explores the path from ‘micro’ to ‘nano’ by using nanotechnology in AP and IP [13]. For example, the incorporation of silver nanoparticles in the polymer matrix or as a coating has proven to add antibacterial functionality to prolong the shelf life of packed foods [14]. AP could play a crucial role in reducing food waste, but should be subject to regulatory approval and consumer acceptance in terms of product safety due to potential migration [15]. Analytical methods for testing the migration of active ingredients are required to adequately detect and quantify the actual consumer exposure levels [16]. So far, the market implementation of AP and IP suffered from a complex cooperation between the stakeholders in the food packaging value chain: industry, consumers, scientists, legislators, etc. [10].

While developing innovative packaging solutions that ensure safe and high-quality foods, the food packaging industry also faces the significant challenge of addressing the environmental impact of conventional plastics. Their extensive use relies heavily on fossil fuels, which leads to the depletion of non-renewable resources and increased carbon emissions. The World Economic Forum estimates that all plastics currently consume 4–8% of global oil, with estimations rising to about 20% in 2050 [17]. In addition, global primary plastic production generated about 5.3% of total global GHG emissions for manufacturing polymers such as PE, PP, and PET in 2019 [18]. Moreover, the widely used traditional oil-based plastics are accumulating in soils, waters, coastlines, and the human body, with geophysical and biological impacts as a result [19]. Worldwide, and especially in the European Union (EU), strategies are being implemented that aim at a circular economy for plastic packaging (e.g., the EU’s Plastics strategy and the Green Deal) [20]. In 2021, plastic packaging waste recycling reached 39.7% [21], but with the upcoming enactment of the Packaging and Packaging Waste Regulation [22], a 55% recycling target for plastic packaging by 2030 is set [23]. This will require the development of new and better collecting and recycling technologies (e.g., for multilayer materials) [7,24].

However, even if developed countries have the most efficient recycling technology, many regions around the world will still lack basic waste management. Therefore, the use of biobased materials produced from alternative feedstocks is still suggested as a short-term opportunity to reduce carbon emissions and decouple packaging materials from the fossil-based economy [25]. Moreover, biodegradable food packaging, which is often contaminated with food, can be disposed of together with food waste, and further processed through composting or organic material recycling [26]. Nevertheless, more sustainable packaging materials must still match or exceed the performance of traditional plastics in terms of protection, shelf life, and cost.

In summary, plastic food packaging is essential in the current food supply chain. Current advancements in packaging technologies, such as multilayers and active systems, can contribute to minimizing packaging material while ensuring food quality and safety, and reducing food waste. However, searching for novel packaging materials is essential to address the End-of-Life (EoL), environmental, and food waste challenges faced by current food packaging. Alternatives, especially biobased and biodegradable mono-materials or bio-nanocomposites, show great potential as more sustainable options.

## 2. Bioplastics: Classification, End-of-Life and Challenges

**Figure 1:**
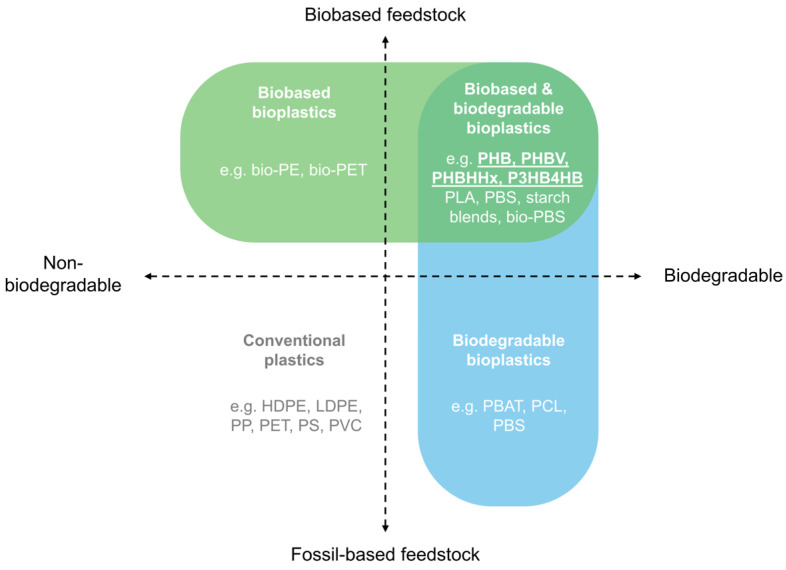
Classification of conventional plastics and bioplastics according to their feedstock origin and potential for biodegradation. The horizontal axis categorizes the materials as biodegradable or non-biodegradable, while the vertical axis categorizes them by feedstock: either biobased or fossil-based. Bioplastics are divided into three main groups: (1) biobased but non-biodegradable plastics such as bio-PE, (2) biobased and biodegradable plastics such as PHAs (PHB, PHBV, and PHBHHx), and (3) fossil-based but biodegradable plastics such as PBAT. Figure redrawn from [27].

Bioplastics include various material types, with different origins and with different EoL possibilities. According to European Bioplastics, plastics are defined as bioplastics if they are biobased, biodegradable, or feature both properties, as illustrated in Figure 1 [27]. Biobased bioplastics are (partly) derived from biomass or renewables, e.g., sourced from corn, sugarcane, canola, soy, plant oil, or various waste streams. Biodegradable plastics are prone to biodegradation, which is a biochemical process where microorganisms break down materials into natural substances like water, carbon dioxide, and biomass without the need for artificial additives. The rate of biodegradation is different in industrial and home composting, soil, water, or marine environments due to the respective temperatures and the presence of the required microorganisms [28].

Biobased plastics can also be classified according to their production method or source as (i) polymers extracted directly from plant biomass, including polysaccharides such as starch or alginates; (ii) polymers synthesized by microorganisms, such as polyhydroxyalkanoates (PHAs), cellulose, xanthan, or pullulan; and (iii) polymers produced through traditional chemical synthesis utilizing renewable biobased monomers, like biodegradable polylactic acid (PLA) and polybutylene succinate (bio-PBS), or durable bio-PET and bio-PE. The term ‘bioplastics’ is lately discouraged for fossil-based biodegradable plastics, like PBS, polybutylene adipate terephthalate (PBAT), or polycaprolactone (PCL) [29].

Rosenboom et al. have also summarized current and future EoL scenarios for bioplastics. The optimal recycling route depends on the bioplastic type, and can include mechanical, chemical, or biological recycling, composting, biodegradation, anaerobic digestion, incineration, or landfill [29]. Bioplastic recycling faces challenges due to the low thermal stability of many biopolymers during re-processing. In addition, a revision of the collection and sorting strategy is necessary. Finally, some concerns exist, because bioplastics can contaminate the conventional plastic waste streams. For example, PLA contamination as low as 1 wt.% in the conventional mechanical recycling stream can affect the end product quality [30].

The global bioplastics production is estimated to grow continuously and significantly from around 2.18 million tons in 2023 to approximately 7.43 tons in 2028 [31], though its share in the total plastics production remains below 1%. Although many bioplastics can be processed via conventional conversion technologies like extrusion and injection molding into packaging materials with a range of mechanical properties, there are five reasons why the implementation of bioplastics is hampered: (i) bioplastics are more expensive to produce than fossil-based plastics due to smaller production scales and the cost-competitiveness of crude oil (*economics*); (ii) manufacturing bioplastics can be less energy-efficient and may have additional environmental impacts from agricultural practices (*efficiency*); (iii) the use of edible biomass for bioplastics is controversial due to its competition with food production, and more efficient use of second-generation biowaste needs to be further developed (*ethics*); (iv) inconsistent labeling, contradicting life cycle assessments, and ‘greenwashing’ lead to confusion among consumers and manufacturers, highlighting the need for better education and global standards (*education*); and (v) recycling systems for bioplastics are underdeveloped, leading to confusion about proper disposal and issues with composting (*EoL*) [29].

## 3. Polyhydroxyalkanoates (PHAs)

### 3.1. PHA Production from Available Feedstocks

Current drivers of the bioplastic market include innovative PHAs. PHAs are a class of biobased and biodegradable polymers produced by microorganisms as intracellular carbon and energy storage compounds, which were extracted and characterized for the first time by Maurice Lemoigne in 1925 [32].

**Figure 2:**
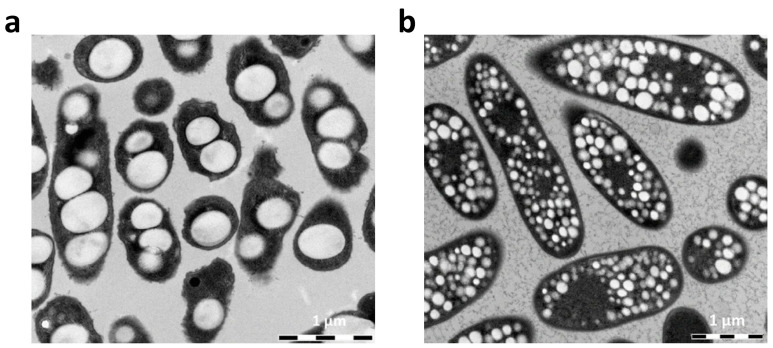
Intracellular synthesis of PHA granules of different sizes in two bacteria: (**a**) *Cupriavidus necator* H16 and (**b**) *Halomonas hydrothermalis* (scale bar 1 µm). Reprinted with permission from Springer Nature [33].

The production of PHAs involves growing microorganisms under specific conditions, typically in the presence of excess carbon sources and limited essential nutrients like nitrogen or phosphorus. Under these conditions, PHA granules with diameters ranging from ~0.2 to 0.5 µm are accumulated intracellularly [33,34]. Examples of PHA granules produced in the cells of the mesophilic bacteria *Cupriavidus necator* H16 and the halophilic *Halomonas hydrothermalis* are shown in Figure 2a and Figure 2b, respectively.

Typically, the accumulated PHA amounts to approximately 30–50% of the dry weight of the bacterial cells, but it can increase up to 80–90%. The molecular weight (MW) of PHAs depends on the growth conditions and type of bacteria and varies between 0.1 and 2.0 × 10^6^ g·mol^−1^. However, by controlling the biosynthesis parameters, ultra-high-MW PHAs with MW > 3.0 × 10^6^ g·mol^−1^ can be produced [35]. PHA biopolymers are mostly extracted from the cells using (halogenated) organic solvents, although more environmentally friendly and solvent-free digestion methods are being developed [29,32,36,37]. After separation and purification, PHA polymers are post-processed to powder or pellets.

**Figure 3:**
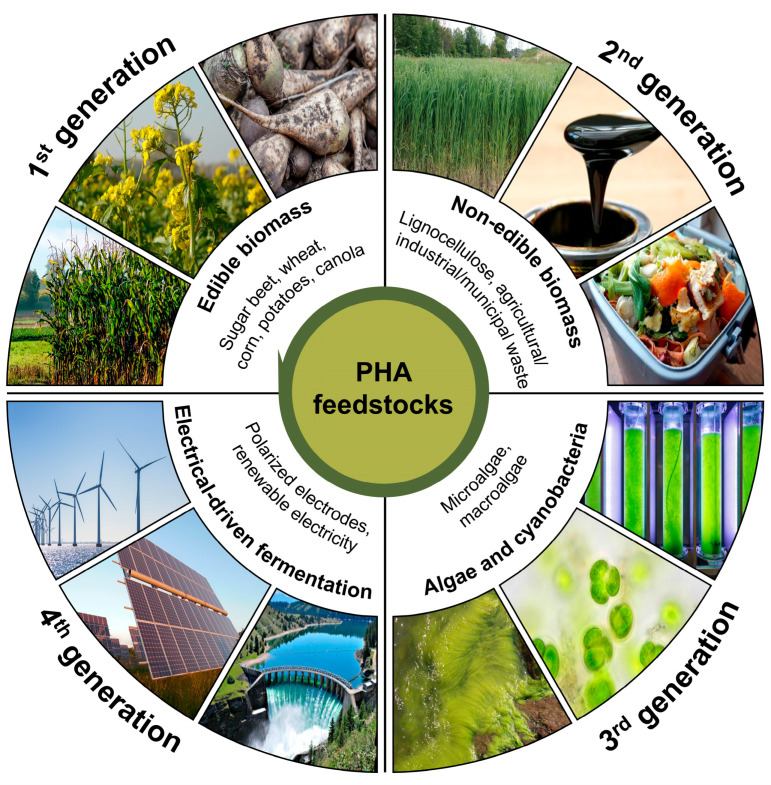
Overview and examples of first-, second-, third-, and fourth-generation feedstocks for the production of PHAs [36,37,39]. First-generation feedstocks consist of edible biomass, while second-generation feedstocks include non-edible agricultural waste and biodegradable municipal waste. Third-generation feedstocks involve algae and cyanobacteria producing PHAs via photosynthesis. Fourth-generation methods, which are still in development, utilize renewable energy sources (wind and solar) for electrical-driven PHA production.

The current production costs of PHAs are approximately 3–4 times more expensive compared to established petrochemical plastics [29]. With the knowledge that ~50% of the PHA production cost is attributed to the organic carbon substrate and culture medium [38], the use of alternative feedstocks has been seriously investigated recently (Figure 3).

First-generation feedstocks are obtained from edible, agricultural raw materials such as corn, wheat, or sugar beets, containing starch or sugars, or plants containing edible triglycerides, like canola oil. Due to their high carbon content, plant oils yield higher PHA amounts per mass than sugar [39]. Second-generation feedstocks are non-edible agricultural by-products like waste starch, cellulose, fatty acids, and organic waste, but also animal by-products or biodegradable municipal waste. The use of waste streams from agricultural, food, municipal, and industrial sources for PHA production does not conflict with arable land use, but can solve waste disposal issues while creating additional profit [39]. Algae are classified as third-generation feedstock for PHA production. In addition to the chemotrophic PHA-producing bacteria, certain cyanobacteria and algae are promising mini-factories, as they can accumulate PHAs by photosynthesis, using light and CO_2_ as primary energy sources, while requiring minimal nutrients for growth. Although still considered in its early stages, the incipient synthesis of PHAs by algae can have important economic and environmental benefits, by reducing production costs, cutting fossil resource use, and reducing CO_2_ emissions [38]. Finally, fourth-generation feedstock processes such as electrical-driven fermentation methods using renewable carbon sources and renewable electricity from wind and solar power to produce PHAs are still in the conceptional design phase [39].

To reduce the production costs, current research in industrial biotechnology is focusing on producing PHAs in open unsterile and continuous fermentation processes using extremophiles (e.g., *Halomonas* spp.), simplifying the downstream processing as well as engineering the PHA biosynthesis in various microorganisms [40,41].

### 3.2. PHA Chemical Structures and Properties

**Figure 4:**
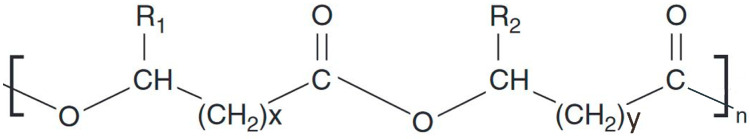
The general chemical structure of PHAs. R_1_ and R_2_ represent alkyl groups or hydrogen, and x and y indicate the number of CH_2_ units.

PHAs are categorized based on the number of carbon atoms in the backbone. Generally, different types of PHAs exist, with short-chain-length (scl) repeating units that contain primarily 3–5 carbon atoms, medium-chain-length (mcl) repeating units that contain 6–14 carbon atoms, long-chain-length (lcl) repeating units with >14 carbon atoms, and copolymers containing scl and mcl repeating units [42]. The general structure of PHAs consists of ester-bonded hydroxyalkanoic acids and is shown in Figure 4. The PHA family exhibits a wide range of mechanical properties, from hard crystalline to elastic. Scl-PHAs are stiff and brittle with a high degree of crystallinity (60–80%), whereas mcl-PHAs and their copolymers are flexible and elastic with lower crystallinity (20–55%), low tensile strength, high elongation at break (300–450%), lower melting temperatures, and glass transition temperatures (T_g_) below room temperature [43]. The most investigated PHAs are poly(3-hydroxybutyrate) (PHB), poly(3-hydroxybutyrate-co-3-hydroxyvalerate) (PHBV), poly(3-hydroxybutyrate-co-3-hydroxyhexanoate) (PHBHHx), and poly(3-hydroxybutyrate-co-4-hydroxybutyrate) (P3HB4HB) (Table 1).

The properties of PHAs, such as mechanical strength, toughness, storage stability, transparency, gas permeability, and biodegradability, are closely related to their crystalline morphology and crystal structure. PHAs will crystallize when the temperature is between the T_g_ and the melting point. Depending on the molecular structure and process conditions, PHAs can form different crystal morphologies, such as single crystals, spherulites (e.g., when crystallized from the melt), or shish-kebab structures (under high shear conditions) [43]. With regard to crystal structures, PHB generally forms α-crystals (with helical chain conformation) during crystallization from the melt or solution, while β-crystals (with zigzag conformation) can be formed by stretching the amorphous phase between the α-crystals [43]. In general, semi-crystalline PHAs suffer from a slow crystallization rate, large crystal size, and easy secondary crystallization. The overall crystallization rate and the tensile strength of PHAs decrease with increasing number of carbon atoms in the (co)monomers content. Adding nucleating agents seems to be the most efficient approach to improving the crystallization rate of PHAs. Moreover, if multifunctional nucleating agents are used, the crystallization rate can be enhanced while simultaneously improving the functionality of the PHA material [43]. In addition, the formation of more β-crystals during processing can substantially improve the strength and toughness of PHAs, while inhibiting secondary crystallization and improving their transparency and storage stability. For example, post-processing, such as uniaxial cold drawing, can increase the orientation of α-crystal domains in PHBHHx (with a rise in overall crystallinity), resulting in higher tensile strength and Young’s modulus with the suppression of secondary crystallization [44]. However, this process is at the expense of material elongation.

Most PHAs are thermoplastic polymers that exhibit a wide variety of mechanical properties rather similar to PE, PS, and PP [42]. PHAs, such as PHB and PHBV, with higher crystallinity and higher melting points, are preferred for rigid applications, while PHAs with lower crystallinity and lower melting points, such as PHBHHx, have more promise in flexible applications [45,46,47]. PHBHHx shows a slower crystallization rate than homopolymer PHB, because the comonomer units are excluded from the PHB lattice during crystallization from the melt [46,47]. In addition, increasing the comonomer content (3-HV or 3-HHx) effectively decreases the crystallinity and, in turn, the melting point and strength of the material, due to the steric hindrance and reduced chain regularity. For example, the melting point and tensile strength of PHBV reduce, respectively, from 175 °C and 45 MPa at 0% 3-HV to 97 °C and 18 MPa at 34% 3-HV [48]. PHA polymer processing is often hampered by their low thermal stability, limiting the processing temperatures and time. For PHB, the melting point is very close to the degradation temperature, which narrows its processing window. For PHBHHx, the higher thermal decomposition temperature compared to PHB and PHBV [46], combined with its lower melting point, creates a favorable processing window for extrusion and injection molding—although the impact of high temperature and shear in melt processing methods such as twin-screw extrusion should not be underestimated [45].

### 3.3. Applications and End-of-Life Options for PHAs

PHAs can be processed using a broad range of industrial technologies, such as injection molding, cast extrusion, (extrusion) blow molding, fiber spinning, and thermoforming [49], enabling many applications. Because of good processability and suitable mechanical and gas barrier properties, PHAs offer a sustainable alternative to conventional (food) packaging materials. The O_2_ permeability coefficient (PO_2_) of PHAs is at least 10× lower compared to conventional plastics such as PP, PE, and PS and relatively close to the PO_2_ of PET and PLA, though still more than 1000 times higher compared to high-barrier materials such as ethylene vinyl alcohol copolymer (EVOH) [42]. The water vapor permeability of PHAs is similar to materials such as EVOH, PET, and PLA, and about 10× higher than more apolar polymers such as PE and PP [42]. Because of their biocompatibility, complete biodegradability, and non-cytotoxicity, PHAs also find applications in biomedical devices, tissue engineering, drug delivery carriers, implants, and cosmetics [36,37]. Due to their biodegradability, PHAs are applied as green films or controlled biodegradable carriers for pesticides in agriculture [36,37]. Moreover, PHAs can be applied as fastmoving consumer goods and textiles [37,50].

The production of PHAs should occur in parallel with the development and implementation of competent recycling routes. Today, it is not clear which recycling methods will have a major impact on PHA waste management, but at least four strategies are being investigated: (i) mechanical recycling, which is feasible if a homogenous input can be obtained and if the loss of properties after multiple re-processing cycles is acceptable to some extent; (ii) chemical recycling through pyrolysis, cracking, or gasification, which aims at converting biopolymers into valuable products, such as chemicals or fuels, opening new applications and markets; (iii) biological recycling via biodegradation in composting, soil or aqueous environments, or (iv) biological recycling via anaerobic digestion toward biogas production [51,52]. Although PHAs are considered to be biodegradable, the time needed for total biodegradation depends on the polymer characteristics (crystallinity, MW, present additives), environmental conditions, and the microbial communities [51,52].

## 4. Zinc Oxide (ZnO) Nanoparticles

Metal and metal oxide NPs such as Ag, Au, TiO_2_, and CuO, and especially ZnO, have gained attraction for use in food packaging applications because of their unique properties, such as size, shape, chemical composition, physiochemical stability, crystal structure, and larger surface area. Incorporating NPs in the polymer matrix can enhance packaging material performance by, for example, reducing bacterial growth, improving barrier and thermal properties, or altering surface wettability and hydrophobicity [53].

### 4.1. Synthesis of ZnO NPs

ZnO NP synthesis methods have been thoroughly reviewed by Goswami et al. [54]. They can be categorized as bottom-up and top-down approaches. The bottom-up approach uses atoms and molecules to create NPs, while top-down approaches cut or slice bulk material to obtain nanosized particles [55]. A wide range of processes have been investigated to produce ZnO NPs with different sizes, shapes, and morphologies, categorized as chemical, physical, or biological methods. Chemical production methods include hydrothermal, sol–gel, solvothermal, microemulsion, and co-precipitation methods [56,57]. Physical methods include microwave and vapor deposition [57]. Current research is exploring green (biological) synthesis of ZnO NPs, while minimizing waste and toxic solvents, and improving energy efficiency, etc. [56]. Green synthesis involves the reaction of zinc salts with biological substrates, followed by the formation of ZnO NPs through thermal treatment [56]. Some specific biological components, such as phytochemicals, can reduce the salt precursors and stabilize the resulting ZnO NPs by acting as capping agents [58,59]. Green ZnO NPs can be synthesized from a wide range of biological sources, including algae, bacteria, fungi, plants, and proteins [60]. This eco-friendly method (e.g., plant-mediated synthesis) offers advantages for various applications, especially in the biomedical field, due to enhanced biocompatibility and the reduced use of toxic chemicals [61]. It is clear that different synthetic pathways yield ZnO NPs with variable morphologies and sizes, which might lead to varying interactions with cells or (bio)polymers.

### 4.2. Application Areas for ZnO NPs

In addition to low cost, good availability, and chemical and physical stability, ZnO NPs also possess unique antimicrobial properties and good catalytic and photochemical activities, with high optical absorption in the UVA (315–400 nm) and UVB (280–315 nm) spectra [62]. Therefore, ZnO NPs are used in a wide range of applications, including biomedicine [63], cosmetics [64], agriculture [65], water purification and disinfection [66,67], and photovoltaics [68,69]. ZnO NPs are classified as generally recognized as safe (GRAS) by the Food and Drug Administration (FDA 2011) US Code of Federal Regulations (Title 21-CFR 182.8991) [70,71]. This has opened many opportunities for the use of ZnO NPs in pharmaceutical drugs, sanitizers, cosmetics, and food packaging.

### 4.3. Antimicrobial Properties of ZnO NPs

**Figure 5:**
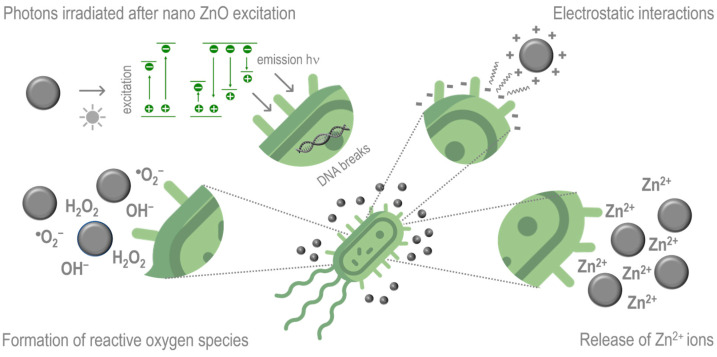
Schematic representation of different mechanisms of ZnO NP antimicrobial activity [72,73,74]. The antibacterial activity of ZnO NPs can be related to direct and indirect interactions, such as electrostatic interactions, the release of Zn^2+^ ions, the generation of reactive oxygen species (ROS), or photons irradiated after ZnO excitation [72,73,74]. The antibacterial efficacy of ZnO NPs can depend on the ZnO NP morphology, composition, or concentration, or the specific growth medium.

Due to its distinctive electronic configuration, ZnO belongs to the novel antibacterial active nanomaterials. Although the exact mechanism of action of ZnO NPs has not been elucidated yet, most studies point to several crucial antibacterial activity mechanisms, including electrostatic interaction between ZnO NPs and microorganisms, the release of Zn^2+^ ions, and the formation of reactive oxygen species (ROS) (Figure 5) [62,72,73]. According to different studies, the microbial inactivation involves the direct interaction between ZnO NPs and the surface of cells, affecting the permeability of the membrane, allowing the internalization of NPs, and inducing oxidative stress, resulting in the inhibition of cell growth. In addition, the release of Zn^2+^ ions and adhesion on the cell surface can also cause mechanical damage to the cell wall and affect bacterial metabolism. The growth of microorganisms can also be hindered when Zn^2+^ ions react with proteins, nucleic acids, and enzymes. Other studies show that exposure of ZnO NPs to UV light can produce ROS such as hydrogen peroxide (H_2_O_2_), hydroxide (OH^−^), and superoxide (•O_2_^−^) anions, which can cause damage to cellular components, such as lipids, proteins, and DNA [62,72]. In addition, the photons irradiated after nano ZnO excitation may also destroy or interfere with the replication of the genetic information of bacteria, thereby inhibiting bacterial reproduction [74].

The controversial antimicrobial efficacy of ZnO NPs may be explained by the fact that the bactericidal mechanism of action depends on specific parameters, such as the morphology, composition, and concentration of the ZnO NPs as well as the media used because the species of dissolved Zn may change according to the medium components [62].

### 4.4. Use of ZnO NPs to Improve Plastic Packaging Performance

ZnO nanocomposites have been widely investigated in conventional plastics like LDPE [75,76], HDPE [77,78], and PP [79,80,81]. In recent years, there has been growing interest in the use of bioplastic ZnO nanocomposites in the packaging industry [57], like PLA [82,83], cassava starch [84], potato starch [85], chitosan [86], and others [87,88]. ZnO NPs have been used as nanofillers to reinforce plastics, i.e., to increase their strength and stiffness. These effects were found to be dependent on the nanofiller characteristics, such as specific surface area, particle geometry, chemical modifications, the potential for ordering in three-dimensional networks, and the formation of polymer/nanofiller interactions [89]. In some cases, the incorporation of ZnO in (bio)plastics has been shown to significantly improve the oxygen and water vapor barrier. Several mechanisms have been discussed to explain the decrease in O_2_ and H_2_O gas permeability in these nanocomposites, including the formation of a tortuous path, crystallinity enhancement, interaction between the polymer and ZnO, and a reduction in the free volume [57,76,90]. In addition, the incorporation of ZnO NPs can also improve the UV barrier and add antibacterial effects to (bio)polymer materials. For example, several studies have shown that ZnO NP-loaded films can effectively preserve a variety of fresh foods, including fruits, poultry, and seafood [91].

Recent concerns about the safety of nanoparticles in food packaging applications have been rising, due to their potential migration from the packaging material into food. NPs have a larger surface area-to-volume ratio compared to their bulk counterparts, which may contribute to their different toxicity [92]. Recent research has shown that ZnO NPs can cause a decrease in cell viability, cellular damage, cytotoxicity and genotoxicity [93], apoptosis, and cytoskeleton changes in human cell lines [94]. However, the exact toxicity of (ZnO) NPs depends on their intrinsic nature, size, agglomeration state, concentration, surface characteristics (e.g., hydrophilicity), migration form, and duration of exposure [95]. For example, it has been shown that migration is reduced when the NPs are completely embedded in the polymer matrix of the food packaging material [96]. In addition, Bott et al. reported that only NPs with diameters below approximately 3.5 nm can cause measurable migration [97]. In the European Union, Regulation (EU) 2016/1416 amending Regulation (EU) 10/2011, on plastic materials and articles intended to come into contact with food, determines which food contact materials (FCMs) can be brought into the European market. ZnO in bulk form is authorized as an additive for FCMs, with a specific migration limit (SML) of 25 mg/kg food, expressed as zinc. The European Food Safety Authority (EFSA) panel on FCMs has stated that ZnO NPs for use as UV absorbers (up to 2 wt.%) do not migrate from unplasticized polyolefins in their nanoform. Therefore, the safety assessment should focus on the migration of soluble Zn^2+^ ions. Though the migration of Zn^2+^ ions from this nanocomposite complied with the current SML for zinc, caution is advised as the upper limit of 25 mg/person per day could be exceeded in combination with dietary exposure from sources other than packaging [98,99].

Finally, regarding the EoL scenario of bioplastic nanocomposites, ZnO NPs can also potentially accelerate the disintegration of bioplastics under hydrolytic or composting conditions, as shown for PLA/ZnO [100,101] or PBAT/PLA/ZnO [102], respectively.

## 5. Conventional and Innovative Technologies to Produce PHA/ZnO Nanocomposites

The method of incorporating ZnO NPs into (bio)polymer matrices determines their dispersion state and crystallinity. This, in turn, affects the final properties of the nanocomposite [103]. Early research on the production of PHA/ZnO nanocomposites has mainly focused on solvent-assisted production methods such as solvent casting and spinning techniques. In this section, we also discuss scalable and novel techniques, such as melt processing, centrifugal fiber spinning, miniemulsion encapsulation, and ultrasonic spray coating.

### 5.1. Solvent Casting of PHA/ZnO Nanocomposites

The most popular method at the lab scale is the solvent casting (SC) technique [104,105,106,107,108,109,110]. This simple but versatile and low-cost technique involves the dissolution of PHAs in organic solvents (e.g., chloroform), followed by sonication in the presence of ZnO NPs, and casting. While representative prototype films can be fabricated, the use of large quantities of volatile (often toxic) solvents, restrictions on film shape and size, and difficulties in controlling temperature and/or humidity are rather disadvantageous with respect to industrial upscaling.

### 5.2. Melt Processing of PHA/ZnO Nanocomposites

**Figure 6:**
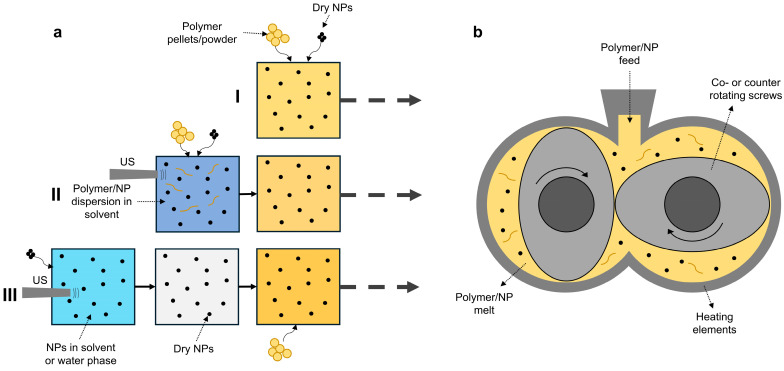
Overview of melt processing methods for the incorporation of ZnO NPs in PHAs. (**a**) Pre-processing steps before melt extrusion, including (I) direct dry-mixing of ZnO NPs with PHA, (II) solvent-assisted pre-incorporation of ZnO NPs in a PHA solution, and (III) ultrasonication to enhance ZnO NP dispersion. (**b**) Twin-screw extrusion of PHA/ZnO mixtures after pre-incorporation.

Melt processing shows more promise due to its applicability on an industrial scale. However, only a few recent studies reported on the fabrication of PHA/ZnO nanocomposites via melt processing such as (twin-screw) extrusion [111,112,113,114,115]. Achieving proper NP dispersion is a huge challenge, due to the high viscosity during melt-mixing compared to processes like SC. The dispersion quality of ZnO NPs in the polymer matrix can be improved by employing pre-processing methods before melt extrusion, as shown schematically in Figure 6a.

Three methods were previously investigated in the literature:

1. The most simple and cost-effective method is direct dry-mixing of ZnO powder and PHA pellets or powder followed by a melt-mixing process (method I in Figure 6a) [116]. It is essential to prepare a homogeneous dry mix of polymer and NPs by using high-speed mixing equipment. Our studies showed that this simple dry-mixing process can result in PHA/ZnO nanocomposites with sufficient dispersion quality [116].
2. A second method involves a combination of solvent-assisted pre-incorporation followed by further dispersion via melt-mixing (method II in Figure 6a) [117,118]. Here, ZnO NPs (mostly at high concentration) are added to a PHA solution in an organic solvent such as chloroform and rigorously stirred (and sometimes sonicated). This dispersion is then casted, and the solvent is evaporated to result in a solid PHA/ZnO ‘master blend’. This material is then shredded and diluted with PHA raw material in the extruder and further mixed in the melt (Figure 6b).
3. A third method includes the combination of an ultrasonication (US) step prior to melt processing to improve the initial dispersion state of ZnO (method III in Figure 6a) [119,120]. Here, a dispersion of ZnO NPs in a solvent or water/solvent mixture (e.g., water/ethanol) is ultrasonicated (US) and dried. This ZnO powder is then dry-mixed with PHA pellets or powder, similar to method I.

After melt-mixing, PHA/ZnO nanocomposites can be further processed using injection molding [45] or into films using compression molding [116,118,121], or they can be directly extruded as films with a die head.

To conclude, melt processing (with or without pre-processing) has the ability to flexibly upscale the production of PHA/ZnO nanocomposites with sufficient dispersion quality. The nanocomposite properties and process efficiency can be improved, e.g., by co-extruding a thin PHA/ZnO nanocomposite layer on top of a PHA film.

### 5.3. Production of PHA/ZnO Nanofibers

#### 5.3.1. Overview of Spinning Techniques

**Figure 7:**
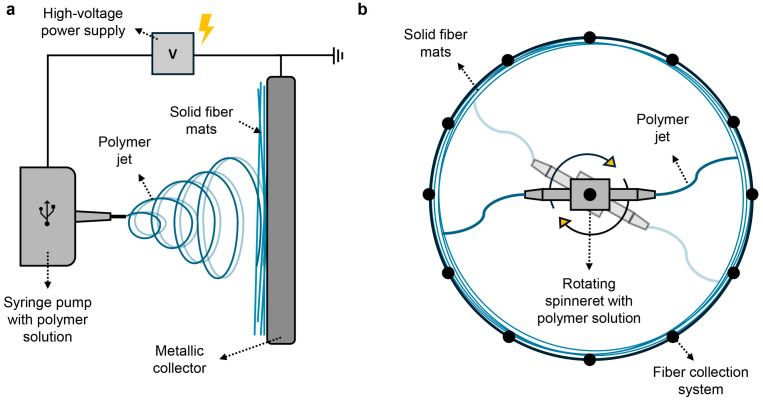
Schematic comparison of (nano)fiber production techniques. (**a**) Electrospinning (ES), where polymer solutions are pumped through a charged nozzle with fiber formation at the metallic collector, influenced by factors such as flow rate and voltage. (**b**) Centrifugal fiber spinning (CFS), where polymer solutions are expelled from a rotating spinneret under the influence of centrifugal forces with fiber formation at the collector system, enabling higher production rates.

Micro- and nanosized fibers have gained significant attention for use in the biomedical sector in tissue engineering, drug delivery, or biosensing, but also in other fields such as water treatment, sensing applications, supercapacitors, and more [122,123]. Several methods are used to produce nanofibers (NFs), including electrospinning (ES), CO_2_ laser supersonic drawing, solution blow spinning (SBS), centrifugal fiber spinning (CFS), phase separation, emulsion spinning, melt spinning, and electrohydrodynamic direct writing [123,124]. Of all the current techniques, ES is the most commonly established technique. In a typical ES experiment, a polymer solution is pumped through a thin capillary nozzle (such as a needle, i.e., the electrode) that deforms under an applied voltage as a narrowing jet in the direction of the counter electrode (metallic collector) [125], as schematically shown in Figure 7a. The solvent evaporates during this path, and solid micro- to nanofibers (mats) are precipitated with high velocity on the collector (counter electrode) [125]. The main ES parameters that influence the final NF morphology are the applied voltage, solution flow rate, needle-to-collector distance, needle diameter, collector type, humidity, and temperature, and the intrinsic polymer solution characteristics such as conductivity, solvent type, and concentration [126,127]. For example, polymer solutions at higher concentrations (and higher viscosity) often produce NFs with a more regular morphology, but with increased diameter.

Despite the versatility of ES, its industrial use is limited because of low production speeds, high cost per gram of fiber, solution conductivity requirements, safety issues, and the need for high voltages [128,129]. On the other hand, CFS (also called rotary jet spinning (RJS) or Forcespinning™ (FS)), is a promising alternative method for the fabrication of fibers due to its simplicity, high rate of fiber production, broad material choice, lower cost for industrial production, and the ability to produce continuous fibers from polymer solutions into enhanced nonwoven structures with roll-to-roll capabilities [128,130,131]. In addition, the concentration of polymer solutions for CFS is twice as large as that for ES, resulting in less solvent usage [132]. In this way, CFS can be technically, environmentally, and economically advantageous over ES. In a typical CFS experiment (inspired by the cotton candy spinning machine), a polymer solution is pumped or loaded into a fast-rotating cup or spinneret and is expelled as a jet under the influence of the centrifugal force to the collector system (e.g., poles). During the flight, the polymer jet thins and the solvent evaporates due to the high surface area, after which solid fibers are formed at the collector system. This process is schematically shown in Figure 7b.

**Figure 8:**
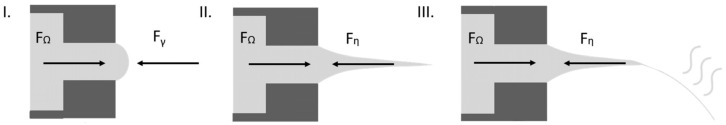
The three stages of a CFS experiment: (**I**) the jet initiation, (**II**) the jet elongation, and (**III**) the solvent evaporation. Adopted from [133].

In more detail, the three stages of CFS include (I) the jet initiation, (II) the jet elongation, and (III) the solvent evaporation (Figure 8).

In the jet initiation stage (Figure 8I), under the influence of a certain rotational speed, a pendant drop is originated from the nozzle orifice, and the rotational forces ($F_{\Omega })$ should be sufficiently high to overcome the capillary force ($F_{\gamma }$) (surface tension) of the polymer solution to form a jet [134]. Once the jet is initiated, the jet elongates under the influence of an interplay between the outward radial centrifugal force ($F_{\Omega })$ and other forces, such as the viscous forces ($F_{\eta }$) of the polymer solution (Figure 8II) [135]. The elongation and stretching of the polymer jet is essential in reducing the jet diameter over the flight distance from the nozzle to the collector [136]. At the end, the polymer jet has thinned extensively, and, due to the high surface area of the jet, the solvent evaporates to form solid fibers (Figure 8III). The solvent evaporation is a binary process that consists of (i) convective removal of the solvent from the jet surface that is dictated by the rotational speed, followed by (ii) solvent diffusion throughout the polymer matrix and evaporation [137]. Research indicates that most solvent evaporation occurs after the fibers land on the collector system [138]. The last stage, therefore, dictates a great part of the final fiber morphology. The final fiber quality, i.e., the formation of continuous, beaded, or beads-on-a-string (BOAS) fibers, is governed by a competition between Rayleigh instabilities and the solvent evaporation rate [137]. But the final polymer fiber morphology is highly dependent on several CFS parameters such as the polymer molecular weight, solution concentration, rotational speed, nozzle diameter and material, and collector distance [133,139,140,141,142].

#### 5.3.2. Production of PHA/ZnO Nanocomposites Using ES and CFS

The processing of PHAs into fibers can be a valuable approach for creating and designing more applications. So far, a variety of synthetic and natural polymers have been electrospun into fibers [143], including PHAs [144] such as PHB [145], PHBV [146,147,148,149], PHBHHx [150,151,152,153,154,155,156], P3HB4HB [157], and blends thereof [158]. After the spinning process, fibers can undergo thermal post-treatments, such as annealing and heat pressing, to produce transparent or translucent films or multilayer structures with excellent mechanical, barrier, and optical properties for food packaging applications [159,160]. Several attempts have been made to develop continuous electrospun-based films from biopolymers, including PHB [161], PHBV [162,163,164], and PLA [165]. Electrospun multilayer structures, such as paper/PHB and PLA [166], and nanocellulose/PHB and PHBV, have also been reported [167]. These electrospun continuous films or multilayer structures show enhanced mechanical and/or barrier properties. In addition, ES has also been used to encapsulate active ingredients to obtain degradable packaging membranes with antimicrobial properties and a high gas barrier [168,169]. Incorporating ZnO NPs can add specific functional properties [145,170,171,172], but can be challenging in case of imperfect dispersion of the NPs in the nanocomposite packaging material [173,174]. Efforts have been made to develop electrospun biopolymer/ZnO nanofibrous composites, such as PHB/ZnO [145,172], PHBV/ZnO [170,171], and PCL/ZnO [175]. In addition, the development of continuous electrospun-based films from PHAs, such as PHBV/copper oxide [176], PHBV/ZnO [104], and PHBV/silver [177], have also been reported.

On the other hand, only a limited number of studies were previously reported on centrifugal fiber spinning (CFS) of PHAs, such as PHB [178,179], PHBV [180], and PHBHHx [181]. Upson et al. [180] showed that the morphology of PHBV fibers depends on the polymer solution viscosity, with the formation of more continuous fibers at higher polymer concentrations (20 and 25 *w*/*v*%), and that their physical–mechanical properties depend on the PHBV concentration, spinning speed, and spinneret-to-collector distance. For PHBHHx, we have demonstrated that the fabrication of continuous PHBHHx fibers with sufficient tensile strength and stiffness and the desired flexibility (ductility) highly depends on the solution viscosity. These findings expand the potential use of PHAs for a wide range of applications [181]. For example, ZnO NPs have been incorporated via CFS into nanofibrous biopolymer composites, such as PHB and PLA, resulting in fiber networks with antimicrobial activity, which could have potential as scaffolding for bone tissue engineering [182,183]. The incorporation of ZnO NPs into PHBHHx fibers via CFS and post-deposition as top layers resulted in PHBHHx/ZnO nanocomposite films with UV barrier properties [184]. Additionally, we have shown the potential of centrifugally spun PHBHHx fibers loaded with dextran NPs for biomedical applications [185].

To conclude, ES and CFS are promising approaches for the production of PHA/ZnO fibers and films with good dispersion quality. The fact that centrifugally spun fiber mats are often loosely packed, compared to the densely packed electrospun fiber mats [128], can make the processing of these fibers into continuous films or top layers a bit more challenging. However, compared to melt processing or solvent casting, the production efficiency (speed) of ES and CFS for the production of packaging films is lower.

### 5.4. Miniemulsion Encapsulation for the Production of PHA/ZnO Nanocomposites

A broad range of materials, including inorganic pigments and other solid NPs, can be encapsulated in a polymeric shell for better mixing in the polymer matrix, for use in functional coatings, and for other applications [186]. Some methods to fabricate inorganic polymer hybrid particles include physical encapsulation (polymer adsorption, layer-by-layer assembly, etc.), and macro-, mini-, and microemulsion polymerization [187]. NPs, such as ZnO, are generally rather hydrophilic, and their encapsulation often leads to non-homogeneous particle morphologies (e.g., due to phase separation), which can be minimized by surface modification of the NPs to improve the compatibility between the inorganic NPs and polymers [188].

#### 5.4.1. Macroemulsion Versus Miniemulsion Polymerization Technique

**Figure 9:**
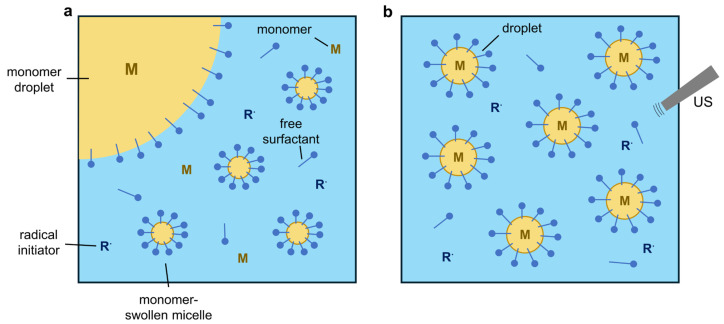
Schematical comparison between the initial particle formation states in (**a**) macroemulsion polymerization, where small monomer-swollen micelles serve as polymerization loci, allowing micellar nucleation and diffusion of monomers to form solid particles, and (**b**) miniemulsion polymerization, where high-energy agitation creates sub-micron droplets (30–500 nm) that act as polymerization loci, resulting in solid particles that ideally match the droplet size. Figure redrawn from [191].

Macro- and miniemulsion polymerization are different in nature regarding their mechanism of particle formation. Macroemulsion is the most widely used particle polymerization technique in both industrial and academic research. Here, particles are formed in the continuous (aqueous) phase via micellar/homogeneous nucleation, while in miniemulsions, the particles are formed via monomer droplet nucleation [189]. The main locus of polymerization in macroemulsions is inside the monomer-swollen micelles, while the locus in miniemulsions is in the monomer droplets [190]. The difference between the formation mechanisms of macro- and miniemulsion polymerization is shown in Figure 9.

In a typical macroemulsion (Figure 9a), the initial particle formation state consists of large monomer droplets acting as reservoirs (>1 µm) and a large number of smaller monomer-swollen micelles acting as the polymerization loci [191]. The micelles act as the polymerization loci because there are many more micelles compared to monomer droplets, so they are more likely to capture the initiator radicals in the aqueous phase (i.e., micellar nucleation) [192]. The monomers diffuse through the aqueous phase from the large reservoirs to the small polymerization particles, after which solid particles are formed.

In miniemulsion polymerization, the monomer phase is broken down into droplets with a sufficiently small size (sub-micron, 30–500 nm [193]) via high-energy agitation via devices such as ultrasonication, as schematically shown in Figure 9b. These monomer droplets can go into direct competition for radical entry to become the primary loci of polymerization [191], compared to the monomer-swollen micelles in macroemulsions.

The sizes of the final solid particles are ideally a 1:1 copy of the monomer droplets [193]. But to achieve a stable miniemulsion, the formed droplets should be stabilized against Ostwald ripening (diffusion process) and coalescence by collisions [194]. The coalescence can be countered by using an appropriate surfactant type, and Ostwald ripening can be minimized by the addition of a specific strong oil-soluble and water-insoluble agent (hydrophobe) [194]. A substantial amount of miniemulsions are performed with the use of the sodium dodecyl sulfate (SDS) surfactant in combination with an oil-soluble hydrophobe, also named ‘cosurfactant’ or ‘costabilizer’ [195]. The drawback of miniemulsion is the use of specialized and energy-intensive equipment (e.g., ultrasonication horn), making it more challenging for use in an industrial context [196]. Due to the specific particle formation mechanism of the miniemulsion process (Figure 9b), i.e., the lack of monomer diffusion through the aqueous phase, it is easily possible to encapsulate a wide range of hydrophobic materials (including inorganic NPs) in the hybrid particles.

#### 5.4.2. The Miniemulsion and Solvent Evaporation (MESE) Technique

**Figure 10:**
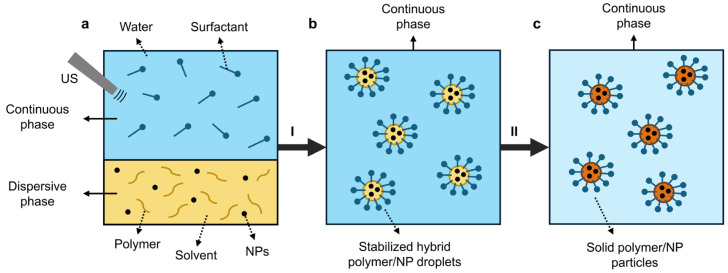
Schematic representation of the miniemulsion encapsulation and solvent evaporation technique (MESE): (**a**) immiscible continuous (aqueous) and dispersive (polymer/NP) phases are ultrasonicated (I) into (**b**) stabilized polymer/NP droplets through surfactant action, and (**c**) solid polymer/NP particles are obtained after solvent evaporation (II). The MESE process parameters, such as oil-to-water ratio, polymer and NP concentration, and surfactant type, significantly influence the final particle size and morphology.

Miniemulsion can be performed to synthesize particles by polymerization of the respective monomers, but also from preformed (bio)polymers [197]. A schematic representation of the miniemulsion and solvent evaporation technique is shown in Figure 10.

The synthesis of encapsulated (nano)particles from preformed biopolymers such as PLA and PHAs starts with the ultrasonication of a dispersive phase containing the polymer and NPs in a specific solvent (e.g., chloroform, cyclohexane, etc.) and a continuous (aqueous) phase containing surfactant (e.g., SDS, polyvinyl alcohol (PVA)). The formed hybrid polymer/NP droplets are stabilized by the surfactant (Figure 10a,b) [197]. After the evaporation of the solvent, solid polymer/NP (nano)particles are obtained (Figure 10c). Several process parameters can influence the final particle size distribution and morphology, including the oil-to-water ratio, polymer and NP amount, polymer MW, and surfactant type and amount. In addition, the stability of the miniemulsions is associated with the avoidance of Ostwald ripening; in the miniemulsion and solvent evaporation process, the polymer and hydrophobic materials can act as hydrophobes to counteract this diffusion process [198].

#### 5.4.3. Production of PHA/ZnO Nanocomposites via MESE

Various (hybrid) particles have been synthesized by the miniemulsion and solvent evaporation technique for a wide range of applications, including synthetic polymers for magnetic optical sensor particles [199] and encapsulated ZnO NPs in PMMA/PS for use in sunscreen formulations [200]. Inorganic NPs have also been incorporated in biopolymers to add functional properties, for example, iron oxide in PLLA [201] or TiO_2_ in PLA [202]. Some studies specifically focused on the fabrication of PHA particles via (modified) (mini)emulsion and solvent evaporation approaches, often with PVA as the surfactant/emulsifier. PHB particles with an average size of 800 ± 300 nm were synthesized by emulsifying a PHB solution in chloroform with an aqueous phase containing PVA surfactant using an ultrasonicator [203]. PHB and PHBHHx particles with sizes ranging from 80–200 nm and 150–300 nm were synthesized via ultrasonication and homogenization methods, respectively, by emulsifying the PHAs in dichloromethane with aqueous solutions of PVA surfactant [204]. PHBV particles in the size range of 253–493 nm were also synthesized via high-speed homogenization (without an ultrasonication step) by emulsifying a PHBV solution in a binary mixture of organic solvents (70/30 *v*/*v*% chloroform/ethanol) and an aqueous phase of PVA surfactant [205]. Other studies showed that MESE to produce PHBV particles can be performed with other surfactants by ultrasonication of PHBV in a chloroform solution with an aqueous phase of SDS surfactant and hexadecane as a costabilizer. Other surfactants, such as Tween-80, have been used to successfully fabricate PHBHHx NPs in the size ranges of 150 nm and 1.5 µm (by changing process parameters) via high-speed homogenization [206].

Despite the efforts to produce PHA-based nanoparticles via the miniemulsion processes, to the best of our knowledge, only limited studies on the incorporation of ZnO NPs in PHAs via the MESE process are yet available. Our recent research showed the possibility of incorporating different types of ZnO NPs into PHBHHx up to loadings of 40 wt.% with particle sizes in the range of 186–231 nm [207].

To conclude, the MESE technique shows promise in fabricating PHA/ZnO particles with excellent dispersion quality. However, current challenges include obtaining large production quantities and reducing high-energy processes such as ultrasonication.

### 5.5. Spray Coating to Produce PHA/ZnO Nanocomposite Films

**Figure 11:**
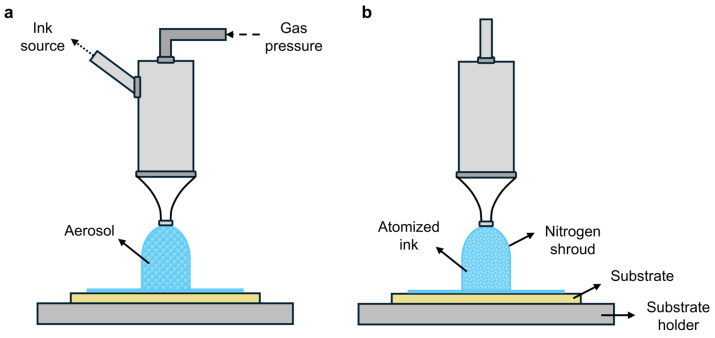
Common spray coating technologies: (**a**) pneumatic spray coating (PSC) uses high-velocity gas to generate small droplets, and (**b**) ultrasonic spray coating (USSC) atomizes the liquid into micrometer-sized droplets, forming a thin layer on the substrate surface. USSC allows the use of water- and alcohol-based solvents, making it suitable for a wide range of materials.

Spray coating is a high-throughput deposition technique for large areas that is widely used for industrial coating applications. There are two commonly available methods: pneumatic-based spray coating (PSC) [208] and ultrasonic spray coating (USSC) [209,210,211]. In the PSC process (Figure 11a), small droplets are generated by a high-velocity gas. An example of PSC is the conventional airbrush [212]. A more advanced technique is USSC, where atomization is realized by mechanical expansion and contraction of piezoelectric transducers in the nozzle. The spraying liquid is spread as a thin layer over the atomization surface of the nozzle and transformed into capillary waves by vibrational ultrasound, which are then converted into a spray of micrometer-size droplets [213]. The droplet size of both methods (Figure 11) is dependent on the nozzle type and specific spraying parameters. The USSC process is schematically shown in Figure 11b. USSC is a rather environmentally friendly coating process due to the possible use of water and alcoholic solvents for depositing a broad range of materials.

PHAs (in particle or solution form) have previously been coated on paper substrates via dip coating [214], blade coating [215], and solvent casting [216] techniques. To the best of our knowledge, no studies are currently available that include the spray coating of PHA materials (in solution or particle form).

Coatings containing ZnO NPs (with or without polymers) have been investigated in the literature via coextrusion [217] and drop-casting [218] for applications including antibacterial activity. Some literature reported the use of PSC (via vaporizer) of other inorganic NPs, such as Al_2_O_3_, SiO_2_, and TiO_2_ NPs, in ethanol on paper to obtain superhydrophobicity [219]. A limited amount of literature is available on the spray coating of ZnO NPs or PHA/ZnO nanocomposites on PHA or other substrates. Abbas et al. investigated ultrasonic spray coating of ZnO NPs (2.5 wt.% dispersion in water/PVA/isopropanol) on PET and PHBHHx substrates for food packaging applications to increase the oxygen barrier properties [220]. No or negligible influence of 50× coating layers of ZnO NPs on PET or PHBHHx was found on the gas permeability, but spraying 3–10 layers of ZnO NPs reduced the UV transmission [220]. Our ongoing research showed the possibility of USSC smooth layers of PHBHHx/ZnO hybrid particles (fabricated by MESE) on PHBHHx substrates with improved UV barrier properties [207].

## 6. Properties of PHA/ZnO Nanocomposites

It has been shown that the incorporation of ZnO NPs into PHA materials can improve or add functional properties. This section summarizes and explains the effects of ZnO NPs on the thermal (crystallization and stability), mechanical, gas barrier, UV barrier, and antibacterial properties as described in the literature. In addition, an overview of available PHA/ZnO studies with a focus on packaging applications is given.

### 6.1. Influence of ZnO NPs on the Thermal Properties of PHAs

A growing body of literature has focused on the development of PHA/ZnO nanocomposites; however, there is no general agreement on the exact influence of ZnO NPs on the processing–structure–property relationships, and specifically on the crystallization behavior of PHAs. Numerous studies have found a delay in the crystallization of PHB, PHBV, and PHBHHx with ZnO NPs, accompanied by a reduction in the crystallinity and/or crystallization temperature (T_c_) [104,111,117,120,145,170,182,221,222]. A reduction in T_c_ with an increase in crystallinity at intermediate ZnO levels was also demonstrated for PHB/ZnO [112]. Other studies did not show notable effects of ZnO NPs on the total crystallinity and crystallization rate of PHB, PHBV, and PHBHHx [113,116,223], with them neither accelerating nor inhibiting the crystallization [114]. On the contrary, ZnO can also accelerate the crystallization of PHB, PHBV, and PHBHHx with a rise in T_c_ and/or with higher crystallinity [105,106,115,118,171,224]. The reported effects were mostly concentration-dependent, diminishing at elevated ZnO NP concentrations due to the formation of agglomerates. The mentioned studies reported acceptable dispersion of ZnO without the need for NP surface treatments, even though improvements in polymer properties are often hindered by poor interfacial compatibility. Only a few studies have been conducted on how surface treatments (e.g., silanization) affect the properties of PHA/ZnO nanocomposites. Silane treatment of ZnO NPs can improve the dispersion quality compared to untreated ZnO NPs, with reduced crystallinity of PHBV or reduced T_c_ of PHBHHx [116,119]. Overall, the exact mechanism by which ZnO NPs influence the crystallization behavior of PHAs is still not clearly understood, and the nucleating effect does not necessarily depend on the dispersion quality. Instead, it can depend on various factors such as the processing method (e.g., solvent casting vs. melt processing), matrix–filler interactions, ZnO NP size, and specific (surface) characteristics.

Regarding the thermal stability of PHA/ZnO nanocomposites, the literature data are still dubious; some studies demonstrated improved thermal stability [105,108,145], whereas others also reported reduced thermal stability [115,116,182]. Improved thermal stability is explained by a good ZnO dispersion quality, strong matrix–filler interactions, which induce a barrier effect against the transport of volatile decomposition products, and high thermal conductivity that facilitates heat dissipation within the nanocomposite matrix [106]. On the other hand, the observed reduced thermal stability of PHA/ZnO can also be explained by the catalytic effect and high heat conductivity of ZnO. A proposed mechanism for the reduced thermal stability of PHA/ZnO nanocomposites is a random chain scission mechanism, with the formation of shorter chain segments and carboxylic terminal groups and crotonic acid as by-products [115]. The formation of zinc salts via a reaction of polymeric carboxylic groups with Zn-OH can further accelerate this degradation process [117].

### 6.2. Influence of ZnO NPs on the Mechanical Properties of PHAs

Several studies have investigated the influence of ZnO NPs (1–10 wt.%) on the mechanical properties of PHB, PHBV, and PHBHHx. An overview of the reported mechanical properties of PHA/ZnO nanocomposites in the literature is shown in Table 2. These nanocomposites were produced using either electrospinning (ES), solvent casting (SC), or melt processing +/− ultrasonication (US). No absolute values of mechanical properties are listed in Table 2, only the changes compared to the reference PHA material without ZnO. A general trend is that ZnO NPs increase the tensile strength (σ) of PHAs. The Young’s modulus (E) of the PHAs generally increases or remains similar to that of the neat PHAs, in combination with a severe reduction in the elongation at break ($\varepsilon_{b}$). Some studies reported increased $\varepsilon_{b}$ values and limited effects on the E [119,120]. In this way, there is no general agreement on the influence of ZnO NPs on the mechanical properties of PHAs. It is important to note that the enhancement of mechanical properties generally tends to diminish at ZnO concentrations above values of approximately 5 wt.% due to the increased possibility of nanoparticle agglomeration. The choice of ZnO NP concentrations ≤ 5 wt.% can be advantageous because it not only reduces costs and minimizes the material opacity, but it could also minimize the risk of ZnO migration to the packed food.

Several literature studies (listed in Table 2) have attributed the increase in strength (σ) and modulus (E) of the PHA/ZnO nanocomposites to strong hydrogen bond interactions between available hydroxyl groups (-OH) on the surface of ZnO NPs and carbonyl groups (C=O) of PHAs. These interactions can enhance the interfacial adhesion between PHAs and ZnO, improving the mechanical properties [105,106,118,225]. However, in the absence (or low amount) of hydroxyl groups on the surface of commercial ZnO NPs, these interactions with PHBHHx cannot be established, thereby minimizing any improvements in mechanical properties [116]. Enrichment of the ZnO NP surface with hydroxyl groups to engage in hydrogen bonding with the polymer matrix can be performed via, e.g., ethanol-assisted sol–gel synthesis [170].

In addition to matrix–filler interactions, the relationship between mechanical properties and crystallinity of PHA/ZnO nanocomposites is often overlooked, even though crystallinity can significantly influence the mechanical behavior. For instance, the incorporation of different ZnO types into PHBHHx (up to 5 wt.%) has shown no significant changes in crystallinity (remaining between ~53 and 56%), corresponding to limited effects on the mechanical properties [116]. In contrast, notable increases in crystallinity from 53% to about 63% and from 55% to about 66% were observed, upon additions of 5 wt.% and 4 wt.% ZnO NPs in PHB [105] and PHBV [106], respectively. This increase in crystallinity was correlated with strongly enhanced strength (σ) and modulus (E) values. This suggests that the influence of ZnO NPs on the mechanical behavior of PHAs may be closely related to changes in crystallinity, although the extent of this enhancement likely depends on the specific interactions between ZnO NPs and the polymer matrix, as well as the ZnO NP dispersion quality.

The different impact of ZnO NPs on the mechanical properties of PHAs can also vary depending on the employed fabrication method (e.g., SC vs. melt processing) [226], as well as the specific ZnO NP characteristics (size, shape, and surface functionalization). In solvent-based processes like SC, the nanocomposite morphology and physical properties can be influenced by factors such as the initial solvent concentration and dispersion quality of the NPs in solution [227], the solvent type [228], and the evaporation kinetics [229]. The mechanical properties of nanocomposites are primarily determined by the final aggregation state of ZnO NPs in the polymer matrix and can, therefore, highly differ between SC and melt processes, making a direct comparison between these methods rather challenging (Table 2). Despite the fact that surface functionalization (e.g., silanization) of ZnO NPs can lead to better dispersion quality, recent studies found that this did not result in further improvements in the mechanical properties of PHBV [119] and PHBHHx [116]. This indicates that factors other than dispersion quality may be more important in influencing the mechanical properties of PHA/ZnO nanocomposites. However, the combination of surface modification (e.g., silanization) and ultrasonication was shown to improve the toughness of PHBV/ZnO, which was related to enhanced dispersion quality and a reduction in crystallinity [119].

In summary, ZnO NPs generally increase the mechanical properties of PHAs, specifically strength and stiffness. Nevertheless, the exact influence of ZnO NPs on the mechanical properties of PHAs remains a complex interplay between specific ZnO characteristics, processing method, matrix–filler interactions, and nanocomposite crystallinity.

### 6.3. Influence of ZnO NPs on the Gas Permeability of PHAs

Surprisingly, only a limited number of studies investigated the oxygen and water vapor gas permeability properties of PHA/ZnO nanocomposites. Results reported in the literature are summarized in Table 3.

Generally, the incorporation of ZnO NPs in PHAs results in a significant decrease in the oxygen and water vapor permeability. In some studies, the improved gas barrier is explained by optimal ZnO dispersion, higher nanocomposite crystallinity, and chain immobilization through matrix–filler interactions [105,106,225]. However, we could not relate the reduced oxygen permeability of PHBHHx/ZnO films to the ZnO dispersion quality, crystallinity, nor to any matrix–filler interactions, but we assume that specific ZnO NP physicochemical properties (e.g., size, porosity, surface area) contribute to the formation of a tortuous path or reduction in the free volume [116], or to the oxygen adsorption capacity of ZnO, which may be influenced by differences in ZnO crystal planes [230]. On the contrary, Castro et al. explained a negative effect on the barrier properties by a decrease in the crystallinity and the higher hydrophilicity of ZnO NPs compared to PHBV [104].

Despite possible improvements in the oxygen gas barrier, the oxygen permeability coefficients of PHA/ZnO nanocomposites are still > 500 times higher compared to high-barrier materials like EVOH [231]. On the other hand, ZnO NPs can significantly enhance the water vapor barrier and reduce the water uptake in PHAs, highlighting their potential for use in protective layers against moisture, e.g., in paper-based applications [166].

### 6.4. Influence of ZnO on the UV Barrier of PHAs

Another practical benefit of adding ZnO to packaging materials is the increase in UV barrier properties. Only some literature studies have reported on the UV barrier properties of PHA/ZnO nanocomposites. Generally, the UV barrier effect is dependent on the ZnO concentration and is enhanced with increasing the ZnO NP concentration. A significant reduction in UV transmission of PHBHHx in the wavelength range of 250 nm to 380 nm has been observed even at low ZnO concentrations of 1 wt.% [116]. Increasing the ZnO concentration up to 5 wt.% further enhances the UV barrier effect, although this compromises the material transparency [116]. The incorporation of ZnO NPs can further reduce the transparency of PHBHHx due to increased crystallinity that is induced by ZnO acting as a nucleating agent [121]. Additionally, ZnO NP coatings on PHBHHx films have also demonstrated a reduction in UV transmission, which intensifies with higher amounts of deposited ZnO NPs [220]. Despite the UV barrier being concentration-dependent, incorporating very high ZnO concentrations up to 30 wt.% can result in diminished UV absorption compared to lower concentrations such as 5 wt.%, due to pronounced agglomeration effects [222].

### 6.5. Antibacterial Activity of PHA/ZnO Nanocomposites

Antibacterial effects of PHA/ZnO nanocomposites have been reported in the literature, against Gram-negative bacteria such as *Escherichia coli*, and Gram-positive bacteria such as *Staphylococcus aureus* and *Listeria monocytogenes* after 24 h (Table 4). Depending on the test method, the activity is mainly reported as log reduction (mathematical expression of the growth inhibition, e.g., a 3 log reduction equals 99% bacteria reduction), growth inhibition (GI, percentage of bacteria reduction), or antibacterial efficiency (AE, e.g., AE = 2 equals 99.99% bacteria reduction—see [145] for equation). These quantitative methods are easier to compare than qualitative methods such as the disk diffusion assay (with a reported inhibition zone) [118]. However, even with quantitative methods, variations in specific test procedures (e.g., surface or immersion testing) and the bacterial strains used can lead to differences in the observed antibacterial efficacy of the nanocomposite materials. Additionally, variations in the concentration of ZnO NPs, the thickness and size of PHA/ZnO films, the chain mobility of the biopolymer, and the ability of ZnO NPs to migrate to the film surface [74] determine the possible direct contact or non-contact antimicrobial mechanisms, as shown in Figure 5. Consequently, the reported antibacterial activities in Table 4 can be challenging to compare.

Based on the reported literature, it is difficult to find a general correlation between the antibacterial activity and the ZnO concentration in PHA/ZnO nanocomposites. Some studies reported better antibacterial activity at relatively low ZnO concentrations (1–5 wt.%), while others reported improved activity at higher concentrations (5–10 wt.%). The enhanced antibacterial activity at high concentrations of 10 wt.% ZnO was explained by the availability of more ZnO at the surface area and, thus, enhanced surface reactivity of the nanocomposites [105]. Furthermore, the antibacterial kinetics have been shown to increase with ZnO NP concentration, from a 1 log reduction at 2 wt.% ZnO to a 3 log reduction at 5 wt.% ZnO in only 3 h [106]. In contrast, some studies reported no further increments of the antibacterial activity by increasing the ZnO NP concentration because agglomeration effects at higher ZnO concentrations reduce the effective reactive surface area [106,145].

Matrix–filler interactions have also been suggested to influence the antibacterial activity of PHA/ZnO nanocomposites. For instance, PHB/ZnO fibers exhibited better antibacterial activity than PLA/ZnO fibers, both prepared by electrospinning, due to weaker matrix–filler interactions [182]. It was hypothesized that stronger matrix–filler interactions (e.g., hydrogen bonding between -OH and C=O groups) may lead to better embedment of ZnO NPs within the polymer fiber, which could, in turn, reduce the antibacterial activity [182]. Interestingly, despite the lower dispersion quality of PHB/ZnO compared to PLA/ZnO, PHB/ZnO still demonstrated superior antibacterial activity, even at lower ZnO concentrations [182]. In PHBHHx/ZnO nanocomposite films, it was shown that intrinsic ZnO characteristics, such as, e.g., the porosity of the NPs, might contribute more to the antibacterial activity than the dispersion state of the nanocomposite [116].

It is obvious that the distribution of ZnO NPs in the nanocomposites might play a key role in the antibacterial efficacy of the PHA/ZnO nanocomposites. For example, the high surface area of electrospun fibers can enlarge the actual contact area between ZnO NPs and bacteria, resulting in different antibacterial activity compared to nanocomposite films with ZnO NPs homogenously mixed in the bulk of the polymer. While to date, no studies have been reported on the coating of ZnO NPs at the surface of PHAs, this could greatly enhance the antibacterial activity, while retaining the (flexible) mechanical properties of the PHAs.

### 6.6. Packaging Performance of Reported PHA/ZnO Nanocomposites

Table 5 summarizes recent studies on PHA/ZnO nanocomposites designed for packaging applications. For each nanocomposite, PHA type, production method, distribution of ZnO in the bulk or as a coating, ZnO NP concentration, and evaluated properties are listed. The proposed applications are indicated as beverage and food containers, blister packages, overwrap, and lamination films [105], but they could also be used for increasing the shelf life of cheese, cereals, and bakery and meat products or for the production of thermoformed containers for fruit juice and dairy products [106]. However, none of the available studies reported on the actual shelf life testing of these food products. Next to pure ZnO NPs, the incorporation of doped or bimetallic ZnO NPs in PHAs for food packaging applications has been investigated, including ZnO:Fe [232] and ZnO:Ag [233,234]. These studies reported antibacterial effects against *P. aeruginosa* [232] or *E. coli* and *S. aureus* [233,234], but are not a focus of this review.

## 7. Limitations and Future Perspectives of PHA/ZnO Nanocomposites

This review demonstrates that PHA/ZnO nanocomposites have great potential for use as active food packaging materials. However, several limitations remain in their implementation. Despite numerous studies that have reported good antibacterial activity against various bacteria (Table 4), none have explored whether this translates to an extended shelf life of food products when applied in practical packaging systems, such as trays or flexible pouches. Such studies are crucial to assess the true potential of these nanocomposites in commercial packaging applications.

Additionally, the safety of PHA/ZnO nanocomposites is not well substantiated in the current literature. There is a lack of migration studies that investigate the release of ZnO NPs into a range of food simulants (according to EU Regulation 10/2011), and the available migration studies do not differentiate between the release of ZnO NPs and Zn^2+^ ions. This new knowledge is essential to ensure food safety and can also contribute to understanding antimicrobial mechanisms of action. New analytical methods to accurately track how ZnO NPs migrate into food under real-life conditions might be useful.

Finally, the impact of ZnO NPs in EoL scenarios, including mechanical, chemical, or organic recycling, remains largely unexplored in the available literature. ZnO NPs could influence these processes either negatively or positively, e.g., by altered crystallinity or thermal stability. Given the current focus on sustainability in packaging materials, this needs to be addressed in the future.

## 8. Conclusions

This paper highlighted the potential of ZnO NPs to enhance the properties of PHAs. The incorporation of ZnO NPs into PHAs can enhance properties like crystallization, mechanical properties, oxygen and water vapor barriers, UV protection, and antibacterial effects. However, the process–structure–property relationships of PHA/ZnO nanocomposites that are reported in the current literature are inconsistent and still not fully understood. We discussed traditional and novel techniques such as solvent casting, melt processing, electrospinning, centrifugal fiber spinning, miniemulsion encapsulation, and ultrasonic spray coating to incorporate ZnO NPs. We identified that the properties of PHA/ZnO nanocomposites are determined by the ZnO NP concentration, intrinsic ZnO NP properties and eventual surface treatments, and processing methods. The combination of these factors determines the complex interplay between ZnO dispersion quality, matrix–filler interactions, crystallinity, etc. Notably, the encapsulation of ZnO NPs within a PHA shell offers significant potential for creating active coatings with enhanced functionality while maintaining material flexibility. Further research regarding the EoL, antimicrobial efficiency, and migration of ZnO NPs in food (simulants) will determine the market potential of PHA/ZnO nanocomposites as sustainable, active, and safe food packaging materials.

## Figures and Tables

**Table 1:** Overview of well-known PHAs illustrating their chemical structure based on the general structure in Figure 4. This classification highlights the structural diversity of the PHA family based on their unique combinations of repeating units.

	R_1_	R_2_	x	y
PHB	-CH_3_	-CH_3_	1	1
PHV	-CH_2_-CH_3_	-CH_2_-CH_3_	1	1
PHBV	-CH_3_	-CH_2_-CH_3_	1	1
PHHx	-CH_2_-CH_2_-CH_3_	-CH_2_-CH_2_-CH_3_	1	1
PHBHHx	-CH_3_	-CH_2_-CH_2_-CH_3_	1	1
P3HB4HB	-CH_3_	-H	1	2

R_1_ and R_2_ represent alkyl groups or hydrogen, and x and y indicate the number of CH_2_ units in the general structure shown in Figure 4.

**Table 2:** Overview of the effect of ZnO NP incorporation on the mechanical properties of PHA/ZnO nanocomposites reported in the literature. Percentual changes with respect to the unfilled PHA material are shown. Symbols are used to indicate an increase (↗), decrease (↘), or no significant change (~) if no absolute values were published.

PHA Type	Production Method	Property	C_ZnO_ (wt.%)	Change (%)
PHB [182]	ES	σ E	1 1	↗ ↗
PHB [105]	SC	σ E $\varepsilon_{b}$	5 10 10	+32 +43 −20
PHBV [106]	SC	σ E $\varepsilon_{b}$	4 4 8	+32 +57 −30
PHBV [120]	Melt	σ E $\varepsilon_{b}$	1 1 1	↗ ~ ↗
PHBV [119]	Melt + US	σ E $\varepsilon_{b}$	1 1 1	↗ ~ ↗
PHBV [223]	ES	σ E $\varepsilon_{b}$	3 3 3	+36 +14 −26
PHBHHx [225]	SC	σ E	5 5	+27 +41
PHBHHx [118]	SC + Melt	σ E $\varepsilon_{b}$	3 3 3	+50 +42 −40
		σ	1	↗
PHBHHx [116]	Melt	E	1	~/↘
		$\varepsilon_{b}$	3	↘

ES—electrospinning, SC—solution casting, Melt—melt processing, US—ultrasonication, σ—tensile strength, E—Young’s modulus, $\varepsilon_{b}$—elongation at break, and C_ZnO_—ZnO concentration.

**Table 3:** Overview of the effects of ZnO NPs on the O_2_ and/or H_2_O vapor permeability of PHA/ZnO nanocomposites reported in the literature. Percentual changes with respect to the unfilled PHA material are shown. Symbols are used to indicate an increase (↗) or no significant change (~) if no absolute values were published.

PHA Type	Production Method	Gas	C_ZnO_ (wt.%)	Change (%)
PHB [105]	SC	O_2_ H_2_O	5 5	−53 −38
PHBV [106]	SC	O_2_ H_2_O	4 4	−35 −46
PHBV [104]	Melt + ES	O_2_ H_2_O	6 6	↗ ~/↗
PHBHHx [225]	SC	O_2_	5	−35
		H_2_O	5	−45
PHBHHx [116]	Melt	O_2_	1 3 5	−36 −23 −26

ES—electrospinning, SC—solution casting, Melt—melt processing, and C_ZnO_—ZnO concentration.

**Table 4:** Overview of the antibacterial activity of PHA/ZnO nanocomposites in literature studies (after 24 h). Maximum antibacterial activities with respect to the unfilled PHA material are shown.

PHA Type	Production Method	Material Morphology	Bacteria	C_ZnO_ (wt.%)	Activity
PHB [182]	ES	Fiber	*E. coli* *S. aureus*	1 1	100% GI 99.99% GI
PHB [105]	SC	Film	*E. coli* *S. aureus*	10 10	97% GI 94% GI
PHB [145]	ES	Fiber	*E. coli* *S. aureus*	3 3	3.20 AE 3.40 AE
PHBV [106]	SC	Film	*E. coli* *S. aureus*	5 5	~99–100% GI ~97–98% GI
PHBV [104]	Melt + ES	Film	*L. monocytogenes*	6	1 to 3 log reduction
PHBV [224]	Laser 3D molding	Scaffold	*E. coli*	5	~60% GI
PHBV [223]	ES + Melt	Film	*E. coli* *S. aureus*	3 3	~>3 log reduction ~>3 log reduction
PHBHHx [225]	SC	Film	*E. coli* *S. aureus*	5 5	98% GI 95% GI
PHBHHx [118]	SC + Melt	Film	*E. coli* *S. aureus*	3 3	~98% GI ~98% GI
PHBHHx [116]	Melt	Film	*E. coli* *S. aureus*	5 5	~26 GI ~72% GI

ES—electrospinning, SC—solution casting, Melt—melt processing, GI—growth inhibition, AE—antibacterial efficiency, and C_ZnO_—ZnO concentration.

**Table 5:** Overview of studies on PHA/ZnO nanocomposite films with a focus on (food) packaging applications. The studies are categorized regarding PHA type, production method, distribution of ZnO in the bulk or as a coating, ZnO concentration, and evaluated properties.

PHA Type	Method	C_ZnO_ (wt.%)	Evaluated Properties
PHB [105]	SC (bulk)	1–10	Thermal, mechanical, gas barrier, water uptake, overall migration, and antibacterial
PHBV [106]	SC (bulk)	1–8	Thermal, mechanical, gas barrier, water uptake, overall migration, and antibacterial
PHBV [104]	Melt and/or ES (bulk and coating)	6	Optical, thermal, mechanical, gas barrier, migration, and antibacterial
PHBV [223]	ES + Melt	1–10	Optical, thermal, mechanical, migration, and antibacterial
PHBHHx [225]	SC	1–5	Mechanical, thermal, gas barrier, water uptake, and antibacterial
PHBHHx [116]	Melt	1–5	Thermal, mechanical, UV barrier, optical, wetting, gas and UV barrier, and antibacterial
PHBHHx [118]	Melt	1–6	Thermal, mechanical, and antibacterial

SC—solution casting, Melt—melt processing, ES—electrospinning, and C_ZnO_—ZnO concentration.

## Data Availability

No new data were created or analyzed in this study.
